# Constitutively Activated DAP12 Induces Functional Anti-Tumor Activation and Maturation of Human Monocyte-Derived DC

**DOI:** 10.3390/ijms22031241

**Published:** 2021-01-27

**Authors:** Robert Dalton, Alexandra Calescibetta, Jun Min Zhou, Michelle Maurin, Grace Ward, Thu Le Trinh, Nhan Tu, Danielle Gilvary, Xianghong Chen, Pingyan Cheng, Elena Kostenko, Sheng Wei, Kenneth L. Wright, Erika A. Eksioglu

**Affiliations:** Department of Immunology, H. Lee Moffitt Cancer Center, Tampa, FL 33612, USA; robert.dalton@moffitt.org (R.D.); alexandra.calescibetta@moffitt.org (A.C.); jun.m.zhou@moffitt.org (J.M.Z.); michelle.maurin@moffitt.org (M.M.); grace.ward@moffitt.org (G.W.); thuletrinh@gmail.com (T.L.T.); nhan.tu@moffitt.org (N.T.); ken1danni2@gmail.com (D.G.); xianghong.chen@moffitt.org (X.C.); pingyan.cheng@moffitt.org (P.C.); elena.kostenko@moffitt.org (E.K.); weishengliu@tom.com (S.W.); ken.wright@moffitt.org (K.L.W.)

**Keywords:** human monocyte-derived dendritic cells, constitutively active DAP12, DC immunotherapy

## Abstract

Dendritic cells (DCs) are professional antigen presenting cells with a great capacity for cross-presentation of exogenous antigens from which robust anti-tumor immune responses ensue. However, this function is not always available and requires DCs to first be primed to induce their maturation. In particular, in the field of DC vaccine design, currently available methodologies have been limited in eliciting a sustained anti-tumor immune response. Mechanistically, part of the maturation response is influenced by the presence of stimulatory receptors relying on ITAM-containing activating adaptor molecules like DAP12, that modulates their function. We hypothesize that activating DAP12 in DC could force their maturation and enhance their potential anti-tumor activity for therapeutic intervention. For this purpose, we developed constitutively active DAP12 mutants that can promote activation of monocyte-derived DC. Here we demonstrate its ability to induce the maturation and activation of monocyte-derived DCs which enhances migration, and T cell stimulation in vitro using primary human cells. Moreover, constitutively active DAP12 stimulates a strong immune response in a murine melanoma model leading to a reduction of tumor burden. This provides proof-of-concept for investigating the pre-activation of antigen presenting cells to enhance the effectiveness of anti-tumor immunotherapies.

## 1. Introduction

Dendritic cells (DCs) mediate the critical interface between innate and adaptive immunity to both microbes and neoplasia [1]. These professional antigen presenting cells (APC) possess the unique capacity to cross-present exogenous antigens derived from tumors to generate both primary and secondary anti-tumor cytolytic responses. In particular, they play a critical role in the development of novel therapeutic strategies against cancer as DCs are among the most potent APCs of the immune system. Hence they represent a feasible, safe, and promising tool in therapeutic vaccination against cancer with minimal side effects and, in some cases, high effectiveness [2]. This last point involves the stage of activation and maturation of the DCs used, making this a critical step for DC vaccine development and one of the major hurdles in their design. In the early stage, immature DCs respond to activating stimuli through a diverse repertoire of stimulatory receptors which direct the maturation, migration, and secretion of critical pro-inflammatory mediators. However, immature cells, from which most DC vaccine therapies are derived, are not migratory or anti-tumoral preventing the migration of tumor-specific cells to the tumor site and elicitation of an effective response [3]. This is partly due to the reduced upregulation or incomplete activation of stimulatory receptors critical for proper DC activation [4]. Many of these stimulatory receptors (such as TREM, Siglec-H, and SIRP-β) associate with Immune-receptor tyrosine-based activation motif (ITAM)-containing adapter molecules through their negatively charged residues in the transmembrane domain to transduce their pro-inflammatory signals to the nucleus [5,6,7].

One such ITAM-containing adaptor molecule is DAP12 (12-kilodalton DNAX activating protein, also known as TYROBP and KARAP) which is expressed by Natural Killer (NK) cells and myeloid cells, including granulocytes, monocytes, macrophages, and DCs. DAP12 mediates signaling for numerous activating cell-surface receptors expressed by these cells [8]. This relatively small 113 amino acid protein maintains a membrane domain and a cytoplasmic tail, containing the canonical ITAM motif YxxL/Ix_6–8_YxxL/I (where x represents any amino acid) [9,10], and where the tyrosine residues within the ITAM domain are both necessary and sufficient for the induction of intracellular signals. Signaling occurs from ligand induced clustering followed by phosphorylation, often by Src family kinases, of the tyrosine residues of the ITAM motifs. Phosphorylated ITAMs create SH2 docking sites, initiating ZAP-70 and Syk kinase signaling to multiple downstream mediators ultimately leading to cellular activation [11]. Functionally, this signaling cascade culminates in antigen directed anti-tumor cytotoxic responses and the regulation of innate and inflammatory cytokine production. Studies have demonstrated that crosslinking of DAP12-associated complexes can lead to myeloid cell activation as determined by enhanced Ca^2+^ influx, MAP kinase activation, and secretion of cytokines and chemokines [12]. Importantly, DAP12 mediated TREM2 signaling is now known to be critical for priming myeloid cell migration, survival, and co-stimulation of the resulting immune response [13,14]. Furthermore, DAP12 has recently been linked to the cross-presentation pathway necessary for the uptake and presentation of antigens derived from apoptotic, necrotic, and conceivably neoplastic cells [15].

Given the crucial role of DAP12 in the inflammatory function of DCs, we hypothesized that we could take advantage of DAP12′s initial signal through the use of a constitutively active form of DAP12 to promote antigen uptake, maturation, migration, and T cell stimulation leading to a more effective anti-tumor immune response. This strategy is meant to avoid accumulation of inactive immature monocyte-derived DCs (Mo-DCs), which could contribute to immune suppression, while inducing phenotypically functional DCs that can migrate to the tumor and induce an active immune response. This approach has been shown by us to be effective in activating accumulated suppressive immature myeloid populations in primary myelodysplastic syndrome (MDS) patient samples [16]. Herein, we fully characterize constitutively active DAP12 mutant constructs and the molecular signaling pathways demonstrating their function in primary Mo-DCs. This work confirms the important role of DAP12-induced maturation, migration, antigen uptake, and T cell stimulation on anti-tumor myeloid cells. Furthermore, we demonstrate here the beneficial anti-tumor immune responses in an in vivo murine tumor model treated with constitutively active DAP12 expressing Mo-DCs. This study provides a novel approach to induce stronger anti-tumor DCs ex vivo for their subsequent use as tumor immunotherapies.

## 2. Results

### 2.1. Constitutively Active DAP12-Mutants Induce Syk Activation in Human Mo-DC

**Figure 1:**
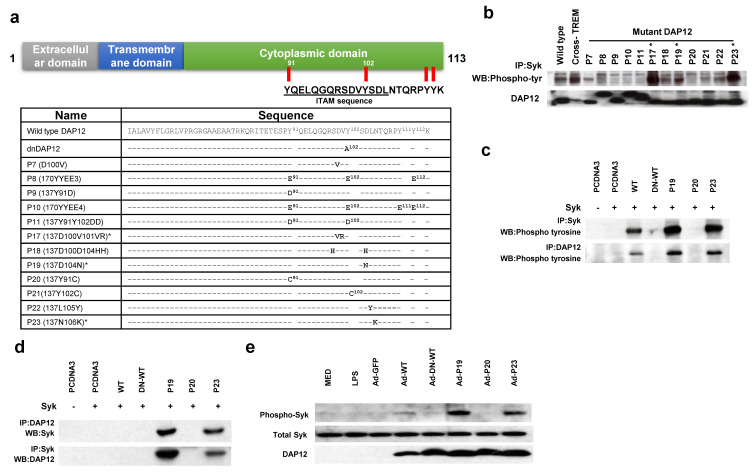
Constitutively active DAP12 associates with and activates Syk in AD293 cells: (**a**) Schematic representation of DAP12 mutations; (**b**) Immunoblot analysis of Syk tyrosine phosphorylation in Syk-overexpressing-AD293 cells transfected with wild-type (WT), dominant negative (DN) DAP12 [21] or mutant DAP12 (P7, P8, P9, P10, P11, P17, P18, P19, P20, P21, P22, and P23) and detected by a pan-phospho-Tyrosine antibody (4G10). Cross-linking of TREM1 in WT DAP12 transfected cells serves as a positive control for Syk activation; (**c**) AD293 cells were co-transfected with pcDNA3-Syk and either WT or mutant DAP12 (P19, P20, and P23) were IP for Syk or DAP12 and WB with anti-phospho-Tyrosine antibody; (**d**) Co-immunoprecipitation of DAP12 and Syk. IP, of either Syk or DAP12, to assess the association of DAP12 and Syk respectively. (**e**) Human monocyte-derived dendritic cells (MoDCs) were transduced with Ad constructs containing either: GFP, WT DAP12, DN-WT, P19, P20, P23, or lipopolysaccharide (LPS) (2 µg/mL) for 48 prior to WB for Syk Tyr525/526, total Syk, or DAP12. Untreated cells (MED) or LPS treated cells are also shown.

We have generated a series of twelve DAP12 mutants within the ITAM domain with the purpose of understanding its function in the maturation of myeloid cells. The chosen sites are conserved amino acid residues within the ITAM domain which are essential for signal propagation [10]. Among the changes are the modification of the tyrosines at positions 91, 102, 111, or 112 into either glutamate or aspartate to mimic phosphorylation [17]. Conversely, 91 or 102 were converted to a cysteine to block activation [18]. We also changed aspartate at position 100 into valine or histidine which are basic amino acids that allow normal binding but with rapid dissociation [19,20], and valine at position 101 into arginine to disrupt the ITAM interaction with a positive charge (Figure 1a). The control dominant negative (DN) form of DAP12 was previously reported by us and is a mutation in the second tyrosine into an alanine shown to not stimulate SYK activation [21].

In order to determine the activity of each mutant DAP12 clone we assessed the activation of Syk, a main signaling molecule recruited by DAP12 through its ITAM domain [22]. For this purpose, overexpressed Syk was immunoprecipitated (IP) from AD293 cells after co-transfection with each of the DAP12 mutants followed by Western blot with a pan-phospho-tyrosine antibody (clone 4G10). As a positive control for Syk activation, antibodies to TREM1 were used to cross-link this receptor and induce signaling [14], bringing Syk and DAP12 in proximity for activation. Of the tested DAP12 mutants, P17, P19, and P23 were able to induce tyrosine phosphorylation of Syk, demonstrating their activation state (Figure 1b). We selected P19 and P23 to continue as p17, as described earlier, is meant to rapidly dissociate, and therefore not constitutively activated. Immunoprecipitation of DAP12 (Figure 1c bottom panel) in these cells revealed higher levels of activation with the P19 and the P23 mutants, compared to WT DAP12, concomitant with Syk activation (Figure 1c, top panel). This observation is consistent with our previous findings in myeloid derived suppressor cells [16]. We then investigated if P19 and P23, physically associates with Syk by co-immunoprecipitating over-expressed DAP12 and Syk. As expected, these activated forms of DAP12 were able to associate with Syk (Figure 1d). Syk, and its downstream pathway, is a novel target for the induction of DC maturation since it is required for the internalization of immune complexes, antigen presentation to T lymphocytes, and IL-12 production after FcRγ engagement [23]. To further characterize if these constructs can induce a functional role in DCs, we expressed the mutant DAP12 constructs in human primary Mo-DCs via an adenoviral vector. Mo-DCs were generated from the peripheral blood of healthy human donors and culturing in GM-CSF and IL-4 followed by transduction with either the WT DAP12, P19, or P23 adenoviral constructs. Flow cytometric analysis of transfected Mo-DC showed that the transduction rates were above 70% as assessed by GFP positivity (Supplemental Figure S1) and that phospho-Syk levels were higher in Mo-DC expressing either Ad-P19 or Ad-P23, compared with WT DAP12 (Figure 1e). These results indicate that the P19 and P23 DAP12 mutants have the ability to initiate the downstream signal without involvement of a surface activating receptor.

### 2.2. Constitutively Active DAP12 Induces Phenotypical Maturation and Activation of Mo-DC

**Figure 2:**
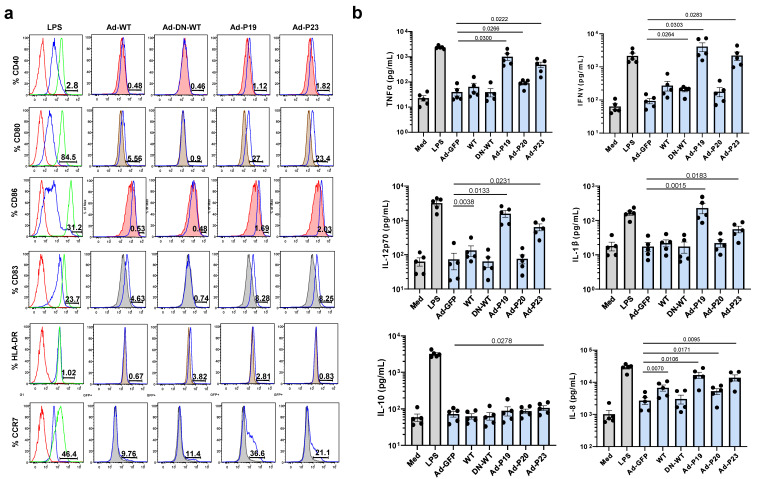
Phenotypic characterization of dendritic cells (DC)differentiation and cytokine profile after transduction with DAP12 mutants. (**a**) Flow cytometric analysis of five-day Mo-DCs were treated with Ad-GFP, Ad-WT, Ad-DN-WT, Ad-P19, Ad-P23, or LPS (2 µg/mL) for 48 h for changes in CD40, CD80, CD86, CD83, HLA-DR and CCR7. Each experimental construct was compared to the empty-vector control, filled histograms, and infected cells were gated on GFP prior to analysis. In LPS histograms, red denotes unstained cells, blue isotype and green is stain as labeled. In subsequent histograms filled histograms are isotype and open are marker of interest as labeled. (**b**) Supernatants from experiment “a” were analyzed by TNF-α, IFN-γ, IL-8, IL-1β, IL-12p70, or IL-10 specific ELISA. Results are expressed as mean value ± SEM of 5 independent experiments. Significance was assessed by comparing against Ad-GFP transfected cells via paired student t test (shown) or ANOVA in Supplemental Table S2. Analyzed data is shown in light blue and medium (MED) and LPS serve as other negative and positive controls respectively (shown in grey).

To determine whether the DAP12 mutants stimulated phenotypic changes associated with DC maturation, flow cytometric analyses were performed on primary Mo-DC from three independent human donors to determine the expression levels of CD83, a specific surface marker of mature DCs, and the co-stimulatory molecules CD40, CD80, and CD86 [24]. We observed an up-regulation of CD80 and CD83 in Mo-DC transduced with Ad-P19 or Ad-P23 compared to those transduced with WT DAP12 vector (Figure 2a and Supplemental Table S1). However, we did not detect changes in the expression of the co-stimulatory molecules CD40 and CD86 with any of the DAP12 constructs. Other hallmarks of DC activation, such as the chemokine receptor CCR7 [25], were also upregulated by both Ad-P19 and Ad-P23. In order to understand if the increase in co-stimulatory protein expression is due to earlier up-regulation of maturation markers by constitutively active DAP12, we also assess expression 24 h after transduction (Supplemental Figure S2). Similar to the 48-h time point, at 24 h P19 and P23 transduced Mo-DCs also show higher CD80, CD83, and CCR7 expression, but with an additional up-regulation of CD86.

Mature DCs are associated with production of inflammatory cytokines and chemokines that define the subsequent anti-tumor response. Hence, we examined the secretory profile of Mo-DC transduced with DAP12 mutants after five days in culture. Ad-P19 and Ad-P23 transduced Mo-DCs secrete significantly higher levels of TNF-α, IFN-γ, IL-12p70, and IL-1β, similar to lipopolysaccharide (LPS)-stimulated Mo-DCs, when compared to either untreated (Med) or Ad-GFP transduced Mo-DC (Figure 2b, Supplemental Table S2). These responses suggest a type I inflammatory response, as constitutively active DAP12 had little effect on IL-10 (Figure 2b) and IL-15 (not shown) secretion. Importantly, IL-8 which is a cytokine strongly linked to mobilization of myeloid cells [26], was also shown to be strongly induced by constitutively active DAP12 (P19 and P23), even above cells overexpressing WT DAP12 transfected cells, suggesting that activation of DAP12 is critical to make MoDC capable of migration after stimulation. Therefore, constitutively active DAP12 constructs can potentially induce a functional type 1 inflammatory and migratory response.

### 2.3. Constitutively Active DAP12 Induces Functional Activation of Mo-DC In Vitro

**Figure 3:**
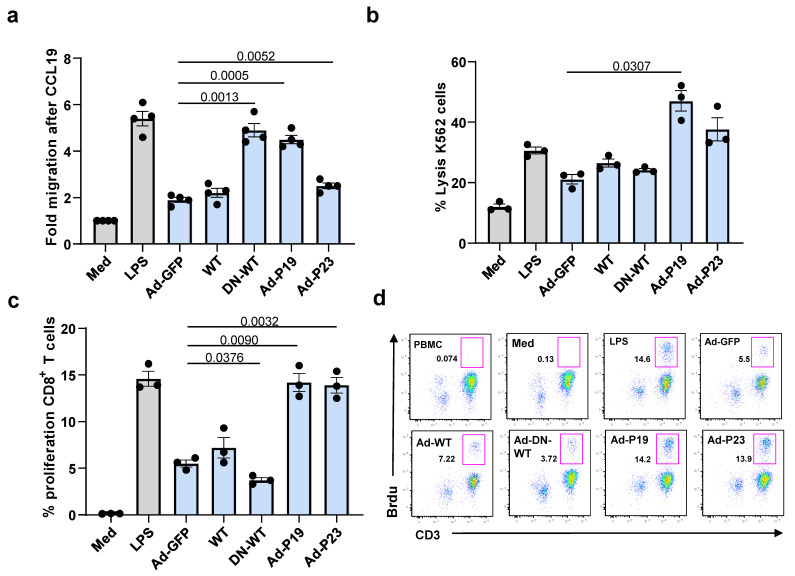
P19 and P23 enhance the migration and activation of Mo-DCs to CCL19 in vitro. (**a**) Migration assay of Mo-DC (5 × 10^4^ cells in 50 µL, top chamber) stimulated with LPS alone (positive control) or in the presence of Ad-GFP, Ad-P19, Ad-P23, Ad-DAP12, or Ad-DN-DAP12 in a micro-chemotaxis chamber. The bottom chambers were filled with serum-free medium with or without CCL19 (100 ng/mL). DC migration rates were determined by counting Diff-Quick stained cells trapped in the filter. The figure represents the fold difference were the experimental control is Med (1) and the background controls is CCL19 untreated cells. (**b**) Mo-DCs as in “a” were pulsed with K562 lysates, treated with mitomycin c and co-cultured with PBMC (2.0 × 10^5^ cells/well). After stimulation, ^51^Cr-labeled target K562 tumor cells were used in a cytotoxicity (^51^Cr-release) assay at an effector:target ratio of 20 to 1. (**c**) The specific proliferation of CD8^+^ T cells in experiment in “b” by BrdU incorporation via flow cytometry. (**d**) Proliferation of total CD3^+^ T cells was measured by Brdu incorporation (representative figure of n = 3). Student t-test analysis is shown. ANOVA analysis p values are in Supplemental Table S2.

Given the fact that Ad-P19 and Ad-P23 up-regulate CCR7 expression on human Mo-DCs, which is associated with enhanced migration toward MIP-3β (CCL19) in vivo [27], and increase the secretion of IL-8 which is linked to migration [26], we examined whether Ad-P19 or Ad-P23 transduced human Mo-DC could migrate toward CCL19 in a chemotaxis trans-well assay. CCL19 induced an increase in migration of LPS-stimulated DCs, Ad-DN-DAP12, Ad-P19, and Ad-P23 transduced Mo-DCs, compared to cells untreated with CCL19 (Figure 3a, Supplemental Table S2). In contrast, exposure to Ad-GFP or Ad-DAP12 only slightly increased the CCL19-induced migration of DCs. Furthermore, when these experiments were performed in the absence of CCL19 there were no significant differences between the migration patterns of transduced DCs indicating that Ad-P19 or Ad-P23 can induce Mo-DC migration in vitro.

To understand if this mobilization is also linked to increased antigen presentation and T cell activation, T cell cytolytic activity was tested by ^51^Cr release assays after stimulation with Mo-DC pulsed with K562 tumor cell lysates. ^51^Cr-labeled target K562 were cultured with CD8^+^ T cells stimulated with pulsed Mo-DC and compared with T cells stimulated with un-pulsed Mo-DC. CD8^+^ T cell stimulated with Mo-DC transduced with Ad-P19 or Ad-P23 showed evidence of antigen-specific lysis of K562 cells (Figure 3b, Supplemental Table S2). CD8^+^ T cell stimulated with Mo-DC transduced with Ad-GFP, Ad-WT, or Ad-DN-WT showed low levels of cytotoxicity against K562 cells. Furthermore, T cells stimulated with Mo-DC in the absence of tumor cell lysates failed to demonstrate lysis of ^51^Cr-labeled targets (data not shown). These data demonstrate that Ad-P19 or Ad-P23 can stimulate Mo-DCs to induce tumor antigen-specific CD8^+^ CTL responses.

In order to demonstrate the ability for these mutant DAP12 constructs to confer DC induced T cell proliferation, DCs were transduced with Ad-GFP, Ad-WT, Ad-DN-WT, Ad-P19, or Ad-P23 for 48 h and examined for their capacity to stimulate the proliferation of T cells. Again, untreated cells (media) served as negative controls while LPS treated cells served as a positive control. P19 and P23 transduced Mo-DC pulsed with K562 tumor lysate induced significant CD8^+^ T cell proliferation (Figure 3c, Supplemental Table S2) as well as overall CD3^+^ positive T cell proliferation after five days co-culture as assessed by BrdU incorporation, when compared to WT control (Figure 3d, Supplemental Table S2). Specifically, we see a doubling of proliferating CD8^+^ T cells, similar to that of LPS stimulated DC. Consistent with the above phenotypical assays, Ad-P19, and Ad-P23 induced the greatest T cell proliferation.

### 2.4. Intratumoral Injection of Constitutively Active DAP12 Transduced DC Induces Significant Antitumor Effects In Vivo

**Figure 4:**
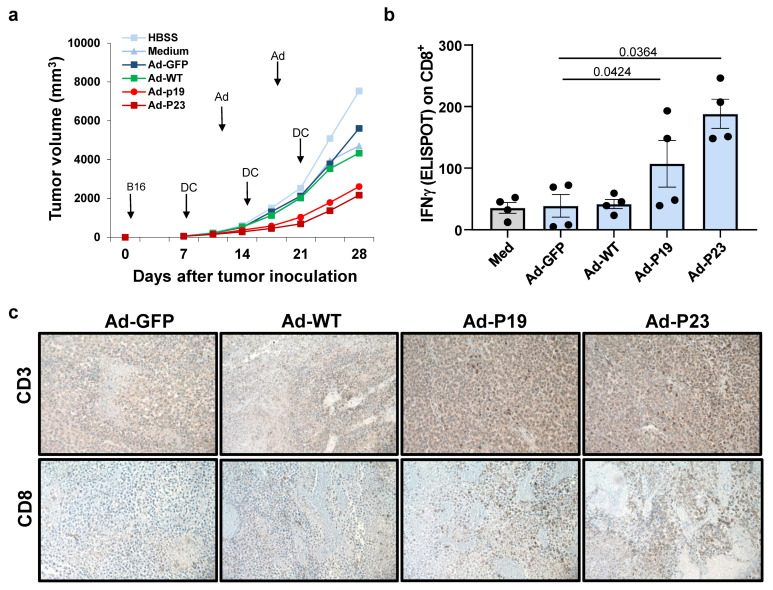
Suppression of tumor growth induced by intratumoral administration of Ad-P23-modified DCs. (**a**) Tumor volume increases (mm^3^, measured twice per week) in murine melanoma model of B16 cells (2.5 × 10^5^) implanted subcutaneously in syngeneic C57BL/6 mice (n = 7/group) that received intratumoral transduced bone marrow DC (Medium-MED, untreated-, Ad-GFP, Ad-WT, Ad-P19, and Ad-P23, 5 × 10^6^ cells per injection) on day 8 and weekly thereafter. Paired student t test was analyzed against all controls (HBSS, Medium, Ad-GFP, blue lines) and WT DAP12 (Ad-WT, green line). Ad-P19 and AdP23 (red lines) were significant against all after the 14-day time point (*p* < 0.05). (**b**) EliSpot detection of IFNγ secretion from CD8^+^ T cells isolated from splenocytes of B16 murine melanoma model as in “a” sacrificed on day 28. The number of spots per 3 × 10^5^ CD8^+^ T cells is shown, and p values were calculated using paired Student’s *t* test. (**c**) Immunohistochemical staining revealed that the number of tumor-infiltrating CD3^+^ T cells (top panels) and CD8^+^ T cells (bottom panels) in the group of mice treated with Ad-P19/DC or Ad-P23/DC was significantly greater than that in the group of mice treated with Ad-GFP/DC, Ad-WT/DC, or DC alone. Panels are representative of samples from 7 mice per group with similar staining profiles and images are displayed at ×200 magnification. Calculated p values of student *t*-test are in Supplemental Table S3 and ANOVA in Supplemental Figure S3b by calculating the AUC.

To examine the in vivo antitumor effect of intratumoral injection of caDAP12 activated bone marrow derived DC we used a subcutaneous murine melanoma model. B16 cells (2.5 × 10^5^) were injected in the right suprascapular area of C57BL/6 mice on day 0 (Figure 4a) followed eight days later by intratumoral injection of DCs alone (medium, MED), treated with HBSS alone, or transduced with Ad-GFP, Ad-WT, Ad-P19, or Ad-P23 (n = 7 mice per group, GFP in Supplemental Figure S3a). Treatment with DC alone (MED), HBSS alone, Ad-GFP DC, or Ad-WT DC did not show significant antitumor effects while significant antitumor effects were observed in mice treated with DC transfected with Ad-P19 or Ad-P23 (Figure 4a, Supplemental Table S3 and Figure S3b).

DCs are antigen presenting cells that play a role in T cell activation, and hence we evaluated the functional activity of CD8^+^ T cells isolated from spleens of the B16 murine melanoma model mice immunized with DCs. C57BL/6 mice (four per group) received 5 × 10^6^ DCs intravenous once a week for three weeks. Seven days after the last immunization, CD8^+^ T cells were purified from pooled splenocytes and IFNγ secretion was evaluated by EliSpot as a measure of activation. We observed that the proportion of the CD8^+^ T cells producing IFN-γ against B16 melanoma cells was significantly greater in CD8^+^ T cells from mice immunized with DC/Ad-P19 or DC/Ad-P23, compared to mice immunized with Ad-GFP/DC or Ad-DAP12/DC (Figure 4b). Substantial evidence suggests that the tumor microenvironment is crucial in tumor progression [28]. In particular, tumor-infiltrating lymphocytes (TILs) have been recognized as principal effectors of the local antitumor immune response. We therefore determined whether Ad-P19/DC or Ad-P23/DC treatment enhanced the presence of CD8^+^ TILs in murine melanoma tumors. Immunohistochemical staining revealed that the number of tumor-infiltrating CD3^+^ T cells and CD8^+^ T cells in the group of mice treated with Ad-P19/DC or Ad-P23/DC was significantly greater than that in the group of mice treated with Ad-GFP/DC, Ad-WT/DC, or DC alone (Figure 4c).

## 3. Discussion

Efficient antigen presentation by DCs is a hallmark of an effective immune response [29]. Despite this, deficient antigen presentation properties are shown by DCs in patients with cancer, which induce a cellular immune deficit that is not intrinsically overcome and leads to a lack of therapeutic responsiveness [30]. As a result, immunotherapy using ex vivo expanded DC is a potential method to give rise to a cellular immune response, in a situation where tumor induced immune suppression is present [31]. However, despite the promise of DC vaccines, where isolated DC are loaded with tumor antigen, little success has been seen in clinical trials [2]. Based on the fact that DC maturation is a necessary first step for these cells to uptake antigen, migrate, and stimulate T cells [32], one option is that ex vivo generated DCs from advanced cancer patients might not have sufficient capacity for inducing tumor antigen-specific immune responses due to the inefficiency of typical DC differentiation cultures (i.e., with GM-CSF and IL-4 or other stimulating cocktails). In the present study we tested the premise that constitutive activation of DAP12 can help override these hurdles for the activation and maturation of DCs, leading to the upregulation of their immune-stimulatory and antigen-presenting capabilities for potential therapeutic use.

Many tumors secrete soluble cytokines that can inhibit the maturation of DC and T cells, and thus suppress the overall immune response [33]. Additionally, in DCs, like other immune cell subsets, there is a unique and complex balance between ITAM and ITIM signaling, and the outcome of such signaling can lead to either cell activation and cytotoxicity, or ineffective cytokine production and death [34]. DAP12 is one such ITAM containing signaling protein that has been shown to be critical for myeloid cell functionality and survival. As a result, we sought new methods to enhance DC activity for adoptive transfer by manipulating this balance using constitutively active mutant DAP12 and demonstrated that transduction of constitutively active DAP12 mutants into primary MoDCs leads to active, functional, and responsive DCs, that induce significant antitumor effects in a B16 murine melanoma model. The introduction of constitutively active DAP12 resulted in improved functionality of DCs, suggesting that DAP12 has activating properties. Our data demonstrated that, unlike wild type DAP12 (Ad-WT) and dominant negative DAP12 (Ad-DN-WT), introduction of constitutively active DAP12 (Ad-P19 or Ad-P23) to immature DCs promote DC maturation, as indicated by up-regulation of CD80, CD83, and CCR7. Interestingly, we found that both Ad-P19 and Ad-P23 were able to induce DC activation including cytokine release, migration, and efficiently increase T cell proliferation and tumor antigen-specific CD8^+^ cytolytic responses. It is important to note, however, that despite the lack of SYK activation dominant negative DAP12 was able to still induce significant migration indicating an alternative migratory signaling effect of our constructs [35,36,37]. This is consistent with the binding of DAP12 to many different receptors and with a variety of functions [8] similar to Syk, a kinase that, apart from self-regulation, can modulate different pathways [38]. Apart from other activated pathways the strength of the interaction between SYK and DAP12 could cause differential effects. Our results hint at that possibility by showing that Syk and DAP12 are phosphorylated in WT-transfected AD293 cells (Figure 1c) but they do not physically interact (Figure 1d), as occurs in P19 and P23-transfected cells. The data could be interpreted as constitutive active DAP12 having a more sustained binding, leading to higher phosphorylation, than the WT transfected cells which may affect the direction of the downstream pathways. Further study will be necessary to understand these effects and their role in Mo-DC vaccination strategies.

DCs have been used to deliver tumor antigen to elicit an antigen dependent immune response to tumor [32]. Since DCs are professional APCs, their ability to process and present antigen is especially important, in a situation where antigen presentation is hampered by tumor induced immune suppression. Ex vivo generation of DCs has involved multiple approaches including peptide loading, differential cytokine support, and adjuvant approaches using toll like receptor ligands [39,40,41,42]. All of the approaches aim to achieve a functional mature DC that will be reinfused into the patient to elicit a potent cellular immune response. However, these vaccines, despite showing early promise, have yet to display significant efficacy in the tumor setting, potentially due to becoming an immature, tolerogenic DC, which inhibit subsequent effector function [43]. Additionally, immature DC, when infused, will not traffic to the tumor like mature DC’s do [44], as fully mature DCs upregulate CCR7 and respond to CCL19 chemotaxis. It is for these reasons we evaluated enhancing DC functionality through an ITAM containing, constitutively active adaptor protein, to facilitate DC maturation into an antigen presenting, anti-tumor DC.

Our group has previously shown that transfection with constitutively active DAP12 stimulates Myeloid Derived Suppressor Cell (MDSC) maturation and improves colony formation in myelodysplastic syndromes [16]. The role of DAP12 in the activation of antigen presenting cell responses has been established [45]. Moreover, early studies in myeloid cells identified DAP12 as a receptor by showing that when ligated by TREM1 it can induce activation and inflammatory responses, particularly of neutrophils and macrophages [46] and the changes in these DAP12 pathways are linked to cancer survival [47]. Additional pro-inflammatory responses have been shown to be amplified by DAP12 in the context of systemic inflammation, where increased cytokine levels were seen [48]. However, unlike MoDC’s, plasmacytoid dendritic cells (pDC) in response to DAP12 signaling with SIGLEC-H down-modulate the large quantities of type I interferon normally present in pDCs and leads to reduced responsiveness to TLR ligands [49]. This is perhaps advantageous in our context as reduction of TLR ligands may also limit the tolerogenic nature of the microenvironment and potentially allow pDCs anti-tumor responses. Future studies with our constructs should be made with analysis of pathways that could disrupt activation of anti-tumor responses into inhibitory/suppressive ones. Gmyrek et al., for instance, recently showed that loss of DAP12 along the FcR gamma leads to acute upregulation of cytokine responses [50] in some subtypes of myeloid cells. However, Mocsai et al. showed similar results to ours shown that SYK activation was reduced in DAP12^−/−^ mice while the role of FcR gamma was only effective at reducing SYK when in combination with a reduction in DAP12 [51]. Based on a comparison on these two studies, and the fact that GM-CSF can modulate myeloid cells [52], we can suggest that pre-activation with GM-CSF, or similar myeloid factors, may lead to myeloid skewing into immature suppressive cells, again suggesting that forced DAP12 may be a novel mechanism to break strong tolerance such as the one induced by the tumor microenvironment. This is critical as it is known that the tumor microenvironment can subvert the expression and functionality of DAP12 in malignancy [53]. Future studies will be needed to demonstrate the basis of this differential role of DAP12 in myeloid cells although its therapeutic potential is already being explored against cancer [54].

In summary, our data suggests that transduction of constitutively DAP12 enhances DC function without inducing adverse events in this preclinical model of subcutaneous B16 melanoma (pathology analysis not shown). Such a modality would represent a significant improvement over other more inflammatory adjuvants, such as TLR ligands [55], or complete/incomplete Freud’s adjuvant [56]. Our work shows that DAP12 activation is both an important mediator of DC activation and maturation, and while the complex nature of ITAM signaling needs additional study, the groundwork laid here suggests that constitutively active DAP12 represents an attractive and potent option for DC vaccine augmentation. The combination of intratumor administration of Ad-P19/DC or Ad-P23/DC can elicit a strong antitumor immune response in vivo, indicating that constitutively activated DAP12 gene therapy could be a useful strategy for directly activating DCs for cancer immunotherapy.

## 4. Materials and Methods

### 4.1. Isolation of PBMCs and Human Monocyte-Derived DC (hMoDC) Preparation

Peripheral blood was purchased as buffy coats from normal healthy donors that donated blood at the Southwest Florida Blood Bank (Tampa, FL, USA). Peripheral blood mononuclear cells (PBMCs) were separated by Ficoll-Hypaque gradient centrifugation (Mediatech Cellgro; Herndon, VA, USA). Cells at the interface were collected and washed three times in cold PBS. After isolation, PBMCs were cultured in 10% FBS RPMI-1640 (GIBCO; Dublin, Ireland) at 37 °C and 5% CO_2_. After 2 h, the non-adherent cells were removed, and the culture plates were washed with cold PBS to obtain a pure fraction of adherent CD14^+^ monocytes. The adherent cells were re-suspended in RPMI-1640 medium supplemented with 10% FBS, 1000 U/mL GM-CSF (Bayer HealthCare, formerly Berlex; Leverkusen, Germany) and 500 U/mL IL-4 (R&D Systems; Minneapolis, MN, USA) in a humidified incubator at 37 °C and 5% CO_2_ for 5 days. Media was replaced every three days while in culture. On day 5, these human monocyte-derived DCs (MoDCs) were further transduced with adenovirus at a multiplicity of infection (MOI) of 50 for an additional 2 days.

### 4.2. Construction of DAP12 Mutants

Two-step overlap extension polymerase chain reaction (PCR) was used to construct DAP12 mutants. Wild-type FLAG-DAP12 and dominant negative FLAG-DAP12 were kindly provided by Dr. Litman (University of South Florida; Tampa, FL, USA) and previously reported [21]. Mutants were created by PCR mutagenic primers using wild-type DAP12 as a template using the primers in supplemental Table S4. P23 was recently reported in a publication [16]. The mutant DAP12 DNA fragments were ligated into the vector PCDNA3 at the Hind III, Xho I restriction sites and cloned into Escherichia coli. Sequence analysis was performed to verify the sequence.

### 4.3. Preparation of Adenoviral Vector

DAP12 mutant plasmids were sub-cloned into a pShuttle-IRES-hrGFP-1 vector (containing the CMV promoter and hrGFP). The PmeI-digested shuttle vectors were then co-transformed into electro-competent BJ5183 bacteria with pAdEasy-1 (containing the viral backbone) and selected on Kanamycin LB plates. The plasmid in the bacteria was amplified and purified using a plasmid maxiprep system (Qiagen; Hilden, Germany). The complete adeno-vector was linearized by PacI digestion and then transfected into AD293 cells using Lipofectamine following manufacturer recommendations (Invitrogen; Carlsbad, CA, USA). The human AD293 cell line was obtained from the American Type Culture Collection (Manassas, VA, USA) and was maintained in Dulbecco’s modified Eagle’s medium supplemented with 10% fetal bovine serum, 100 U/mL penicillin, and 100 µg/mL streptomycin at 37 °C in a humidified 5% CO_2_ incubator. All recombinant adenoviruses were amplified in AD293 cells. Viral stocks were obtained by amplification of the AD293 cells followed by standard two-step CsCl gradient ultracentrifugation, dialysis, and storage as a glycerol (10% volume/volume) stock at −80 °C. The titer of each viral stock was routinely 10^11^–10^12^ plaque forming units (pfu) when assayed on AD293 cells.

### 4.4. Crosslinking, Immunoprecipitation, and Western Analysis

AD293 cells (a cell line derived from HEK293 with improved cell adherence and plaque formation, Agilent; Santa Clara, CA, USA) were washed with Dulbecco’s PBS (DPBS), and resuspended in 2.0 µg/mL of anti-human TREM-1 antibody (R&D Systems; Minneapolis, MN, USA) on ice for 30 min. After washing with DPBS, cells were incubated with 1.0 µg /mL of Affinipure rabbit anti-mouse IgG (H + L) Ab (Jackson ImmunoResearch; West Grove, PA, USA) at 37 °C for 10 min. Cells were then scraped off the plates and followed by centrifugation at 400 g for 10 min at 4 °C. Cells were washed twice with cold 1× DPBS and lysed on ice for 30 min at 4 °C in 1 mL of lysis buffer (1% NP-40, 10 mM Tris, 150 mM NaCl, 0.5 mM phenylmethylsulfonyl fluoride, 10 mM iodoacetamide, 50 mM NaF, 1 mM ethylenediamenetetraacetic acid, 1 mM sodium orthovanadate, 0.25% sodium Deoxycholate, 10 µL Ala-Ala-Phe p-nitroanilide (SIGMA; St. Louis, MO, USA), 10 µL protease cocktail I (SIGMA; St. Louis, MO, USA cat# P2850), and 10 µL protease cocktail II (SIGMA; St. Louis, MO, USA cat# P5726). Cell lysates were centrifuged at 12,000× *g* at 4 °C for 15 min to remove nuclei and cell debris, after which the clarified lysate supernatant was isolated. The protein concentration of the soluble extracts was determined using the Bradford protein assay (Bio-Rad, Hercules, CA, USA) following manufacturer protocol. Western blots were blocked in PBST containing 4% BSA for 1 h and then incubated overnight at 4 °C in primary antibodies. Mouse anti-flag (M2) was purchased from Sigma, phosphotyrosine (clone 4G10) was purchased from Upstate Biotechnology (Lake Placid, NY, USA). Rabbit Abs to phospho-ERK (Thr^202^/Tyr^204^), phospho-Syk (Tyr^525/526^), pan-ERK and pan-Syk were purchased from Cell Signaling Technology. Blots were washed three times in PBST, followed by incubation for 1 h with horseradish peroxidase-conjugated secondary antibodies in PBST containing 5% non-fat dried milk. The blots were washed three times in PBST followed by detection with enhanced chemiluminescence detection system (ECL, Amersham, Piscataway, NJ, USA). In some instances, blots were stripped in stripping buffer (Pierce Biotechnology; Rockford, IL, USA) at 37 °C for 15 min. For IP, cleared lysates with equivalent amount of protein were incubated with 5 µg of Myc-Tag antibody (SIGMA; St. Louis, MO, USA) with overnight rotation at 4 °C, then 30 µL of protein G-agarose beads (SIGMA; St. Louis, MO, USA)) were added, and allowed to mix for 2 h at 4 °C. The precipitates were pelleted by centrifugation, the supernatant was removed, and the pellet washed three times with lysis buffer, and proteins were eluted by heating beads in 50 uL of 2X SDS sample buffer for 10 50 °C. Following pelleting by centrifugation, the supernatant was transferred to a new microcentrifuge tube, and DTT was added at 100 mM. Then, 50 uL of 3X SDS sample buffer with DTT was added to the pelleted beads, constituting a second elution. Following a 5–10 min boil of samples, proteins were resolved by 10% SDS–polyacrylamide gel electrophoresis and transferred to polyvinylidene difluoride (PVDF) membranes for Western blotting.

### 4.5. Flow Cytometry

Dendritic cells were harvested, and 1 × 10^6^ cells were washed with cold 1× DPBS and then incubated with anti-CD40-APC (BD Biosciences; San Jose, CA, USA), anti-CD80-PE (eBioscience; San Diego, CA, USA), anti-CD83-PE-Cy5 (BD Biosciences; San Jose, CA, USA), anti-CD86-PE (eBioscience; San Diego, CA, USA), anti-HLA-DR-PE (eBioscience; San Diego, CA, USA), and anti-CCR7-PE-Cy7 (BD Biosciences; San Jose, CA, USA) labeled antibodies (BD Biosciences; San Jose, CA, USA) for 30 min on ice. After washes, cells were fixed with 1% paraformaldehyde (Fisher Scientific; Waltham, MA, USA) and data were collected on an LSRII flow cytometer (BD Biosciences; San Jose, CA, USA). Expression of cell surface markers was analyzed on gated GFP positive cells using FlowJo 6.3.4 software (FlowJo; Ashland, OR, USA).

### 4.6. DC Migration Assay

Day 5 DCs were transduced with DAP12 adenovirus for 48 h, cells were washed to remove unbound virus, and assayed for migration in response to the chemokine CCL19 (100 ng/mL). Assay medium (25 µL) containing serum-free medium or chemokine CCL19 (100 ng/mL), was loaded into the lower chambers of a trans-well plate. Fifty microliters of DCs suspension (1 × 10^6^/^mL^) in plain RPMI 1640 medium was added to the upper compartment of the chemotaxis chamber. The two compartments were separated by a 5-µm pore size polycarbonate filter (Nucleopore Corp.; Pleasanton, CA, USA). Spontaneous migration was determined as the movement of cells toward the control medium. The chamber was incubated for 1 h at 37 °C in humidified 5% CO_2_ incubator. After culture, the filter was removed and stained with Diff-Quik (Harleco; Gibbstown, NJ, USA). The number of DCs from the upper chamber that infiltrated across the filter to appear on the underside was recorded in three oil immersion fields for each well, and each experimental condition was assayed in triplicate wells.

### 4.7. Cytotoxicity Assays

CD8^+^ T cell were isolated from healthy volunteer PBMC using RosetteSephuman CD8^+^ T cell enrichment cocktail (STEMCELL Technologies; Vancouver, Canada). These cells were then stimulated in vitro with K562 lysate-pulsed DCs (DC:T cell ratio 1:20). At day 6, CD8^+^ T cell were washed extensively in PBS and used in 5 h ^51^Cr-release assay. Briefly, target tumor cells were labeled with 200 μCi of ^51^Cr in 0.2 mL of medium at 37 °C in a 5% CO_2_ atmosphere for 1 h. The cells were then washed three times and added to effector cells at concentration of 5 × 10^3^ cells/well in 96-well round-bottomed culture plates, resulting in E/T ratios ranging from 20:1 to 10:1 in a final volume of 0.2 mL in each well. After 5 h incubation at 37 °C, 100 μL of culture supernatants was harvested and counted in a gamma counter. The percent specific ^51^Cr release was determined according to the equation ((experimental cpm—spontaneous cpm)/total cpm incorporated) × 100. All determinations were done in triplicate, and the SEM of all assays was calculated.

### 4.8. T Cell Proliferation Assays

Day 5 DCs were either transduced with DAP12 adenoviruses or LPS followed by a pulse with K562 lysate. After harvest, DCs were treated with mitomycin C for 1 h at 37 °C, then cells were washed 3 times with 1× DPBS and counted. Cells were plated in a 48-well plate at a 1:20 DC: PBMCs cell ratio (2.0 × 10^5^ PBMC cells/well). After 5 days co-culture, cells were harvested, washed, counted, and analyzed by flow cytometry. To measure proliferation by BrdU incorporation, 10 µM BrdU was added to cell cultures during the final 30 min, and then cells were harvested, stained for CD3, washed, fixed, and permeabilized, then incubated with 300 µg/mL DNase for 1 h at 37 °C. After washing, cells were incubated with APC-conjugated anti-BrdU antibody for 30 min at room temperature and analyzed on a LSRII flow cytometer (BD Biosciences; San Jose, CA, USA).

### 4.9. Antitumor Effects

B16 tumor cells (2.5 × 10^5^) were injected subcutaneously in the right suprascapular area of C57BL/6 mice, and seven-day-old established tumors were treated with an intratumoral injection of 1 × 10^6^ DCs infected with adenovirus in a volume of 100 µL of HBSS administered once a week for 3 weeks. Treatment groups included MED (untreated)/DC, Ad-GFP/DC, Ad-WT/DC, Ad-P19/DC, Ad-P23/DC or HBSS alone with each group consisting of seven animals. Mice received 1 × 10^7^ PFU of adenoviral vector or HBSS as a control on days 13 and 20. Two bisecting diameters of each tumor were measured with calipers, and tumor size was measured every 3–4 days. The volume was calculated using the formula V = 0.4ab^2^ with “a” as the larger diameter and “b” as the smaller diameter. The total length of the study was 28 days.

### 4.10. ELISA and EliSpot Assays

Human MoDC (1 × 10^6^ cells/mL) were transduced with DAP12 adenoviruses or LPS for 48 h. Supernatants were collected and analyzed for the presence of IL-12p70, IL-10, TNF-α, IFN-γ, and IL-1β by ELISA (Ready-SET-Go kit) following the manufacturer’s instructions (eBioscience; San Diego, CA, USA). ELISA kit for IL-15 were purchased from BD Biosciences. For detection of CD8^+^ T cells secreting IFN-γ, EliSpot was performed using CD8^+^ T cells from spleens that were purified by positive selection using antibody-coated magnetic beads following the directions provided by the vendor (Miltenyi Biotec; Auburn, CA, USA). Responder (CD8 purified) cells were incubated at 3 × 10^5^, 1 × 10^5^ and 3 × 10^4^ cells per well together with 5 × 10^4^ stimulator cells (B16 cells). Cultures were incubated at 37 °C for 20 h and spots (IFN-γ producing cells) were developed as described by the EliSpot kit manufacturer (Mabtech, Inc.; Mariemont, OH, USA). Spot counting was done with an AID EliSpot Reader System (Autoimmun Diagnostika GmbH; Strassberg, Germany).

### 4.11. Immunohistochemistry Staining

Formalin-fixed, paraffin-embedded tumor tissues were cut into 5-μM sections. Slides were stained using a Ventana Discovery XT automated system (Ventana Medical Systems; Oro Valley, AZ, USA) as per manufacturer’s protocol with proprietary reagents. Briefly, slides were deparaffinized on the automated system with EZ Prep solution (Ventana Medical Systems; Oro Valley, AZ, USA). Heat-induced epitope retrieval method was used in Cell Conditioning solution (Ventana Medical Systems; Oro Valley, AZ, USA). To block endogenous peroxides and protein the Ventana Discovery XT Inhibitor CM was added to the slides. A rabbit polyclonal primary antibody that reacts to CD3 (Cell Marque; Rocklin, CA, USA) was used at a prediluted strength and incubated for 32 min. The Ventana Anti-rabbit secondary Antibody was used for 20 min. A rabbit monoclonal primary antibody that reacts to CD8 (Abcam; Cambridge, MA, USA) was used at 1:25 and incubated for 60 min. The Ventana Anti-rabbit secondary Antibody was used for 16 min. The detection system used was the Ventana ChromoMap DAB kit and slides were then counterstained with hematoxylin. All of these were performed automatically by the system. Slides were then dehydrated and coverslipped as per normal laboratory protocol.

### 4.12. Generation of Murine Bone Marrow-Derived DC and Transduction with DAP12 Adenovirus

Male C57BL/6 mice aged from 6 to 8 weeks were purchased from NCI-Harlan (Fredrick, MD, USA). All mice were housed in pathogen-free units of the Division of Comparative Medicine at H. Lee Moffitt Cancer Center, University of South Florida (Tampa, FL, USA). Bone marrow MNCs were isolated from the tibias and femurs of C57BL/6 mice and the red blood cells were lysed. DCs were isolated using microbeads and LSMACS columns according to the manufacturer’s protocol (Miltenyi Biotec; Bergisch Gladbach, Germany). Purified DCs were maintained at 37 °C in 5% CO2-humidified atmosphere using RPMI 1640 supplemented with 10% fetal bovine serum (FBS), 20 ng/mL murine recombinant GM-CSF, and 10 ng/mL IL-4. Cell cultures were incubated for 6 days with a media change every 2 days. Recombinant murine IL-4 was purchased from RDI and murine GM-CSF was purchased from MyBiosource Inc. (San Diego, CA, USA).

On day 6, BMDCs were transduced with adenovirus at a multiplicity of infection (MOI) of 200. Briefly, BMDCs were seeded in 2% FBS, antibiotics-free RPMI containing the desired number of viral particles in a final volume of 200 µL. The plates were incubated overnight at 37 °C followed by culturing in complete culture medium with the cytokines for an additional 24 h. BMDCs were harvested, washed with HBSS three times, and removed dead cells using Dead Cell Removal kit (Miltenyi Biotec; Auburn, CA, USA) prior to receiving intravenous (i.v.) injection. In control groups, mice received BMDCs that did not undergo transduction.

### 4.13. Immunizations

Day 6 BMDCs were transduced with MED, Ad-GFP, Ad-P19, Ad-P23, or Ad-DAP12, and the next day they were pulsed with B16 lysate for 24 h. After DCs were harvested, dead cells were removed using Dead Cell Removal kit (Miltenyi Biotec; Auburn, CA, USA). For DCs immunizations, B6 mice were injected intravenously with a 2.5 × 10^6^ treated DCs in a volume of 300 µL once a week for 3 weeks. CTLs for Elispot assays were obtained from DCs-immunized mice 7 days after the third immunization.

### 4.14. Statistical Analysis

Statistical significance to assess the numbers of antigen-specific CD8^+^ T cells (ELISPOT) and cytokine levels (ELISA) were determined by unpaired Student *t* tests as well as one-way Analysis of Variance (ANOVA). Tumor sizes between 2 populations throughout time and cytotoxicity assays at various E:T ratios were analyzed for significance using two-way ANOVA. Additionally, area under the curve (AUC) analysis was conducted on the data from Figure 4a. A one-way ANOVA was applied to these data to compare tumor size throughout time between various treatment groups. All analysis and graphics were done using GraphPad Prism, and *p* values < 0.05 were considered to be statistically significant.

## Data Availability

Data is contained within the article and supplementary material.

## Supplementary Materials

The following are available online at https://www.mdpi.com/1422-0067/22/3/1241/s1.

## Abbreviations

DC	Dendritic Cells
APC	Antigen presenting cells
ITAM	Immunoreceptor tyrosine-based activation motif
DAP12	12-kilodalton DNAX activating protein
NK	Natural killer cells
Mo-DC	Monocyte-derived DC
WT	Wild type
WB	Western blot
IP	Immunoprecipitation
