# Supplementary material for: Constitutively Activated DAP12 Induces Functional Anti-Tumor Activation and Maturation of Human Monocyte-Derived DC
Source: Int J Mol Sci. 2021 Jan 27;22(3):1241. doi: 10.3390/ijms22031241 (PMC7865632; doi:10.3390/ijms22031241)
Supplement: Supplementary file 1 [file ijms-22-01241-s001.pdf]

Supplemental Figure S1

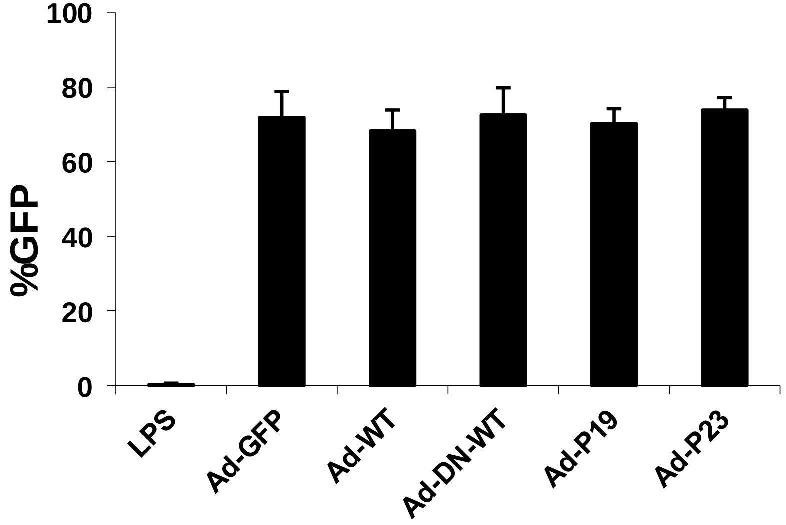

Flow cytometric analysis of GFP expression in transduced primary Mo-DC transduced with adenovirus as shown. LPS is the positive control for the experiment not transduced.

Supplemental Table S1. The effects of Ad-P19 or Ad-P23 on Mo-DC surface marker expression

	Percentage of GFP+ iDC coexpressing indicated markers					
	CD40	CD80	CD83	CD86	HLA-DR	CCR7
	%	%	%	%	%	%
Ad-LPS	6.7±4.8	75.8±15.2	16.9±9.3	24.1±15.0	0.4±0.5	50.2±5.0
Ad-WT	1.3±0.9	5.4±0.3	3.8±2.0	0.8±0.2	0.6±0.4	6.5±3.7
Ad-DN-WT	1.6±1.5	2.5±2.8	1.1±0.6	0.7±0.2	2.1±1.6	6.9±5.1
Ad-P19	2.5±1.2	**25.6±2.3**	**8.1+1.6**	1.5±0.9	1.4±1.2	**28.9±12.8**
Ad-P23	4.0±2.1	**23.1±0.6**	**8.0±2.0**	1.8±1.3	0.8±0.4	**26.4±8.1**

Comparative analysis +/- standard deviation of flow cytometric analysis of different markers in 5 separate experiments. Primary Mo-DC transduced with adenovirus as shown. LPS is the positive control for the experiment transduced with empty GFP+ vector.

Supplemental Figure S2

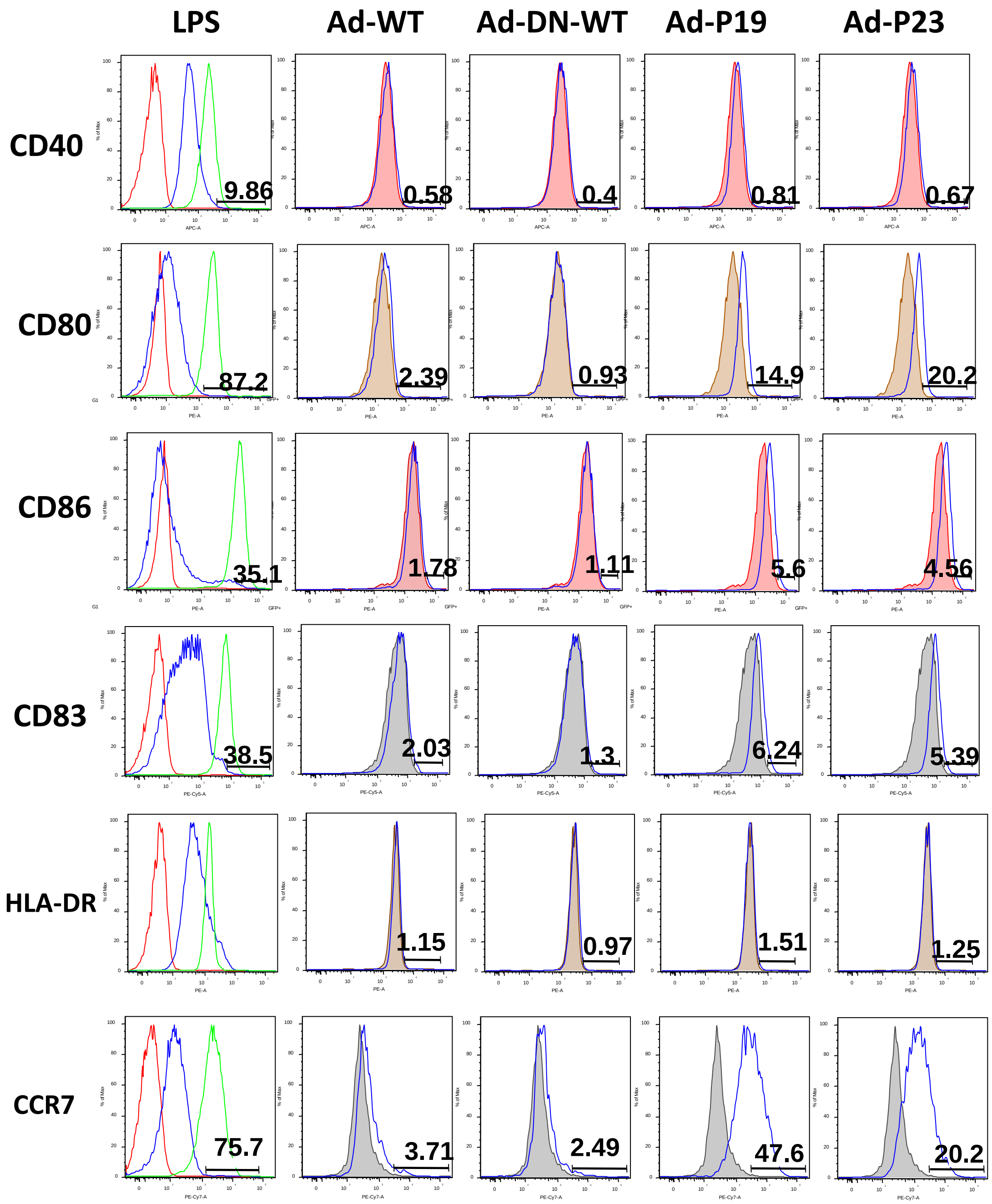

Flow cytometric analysis of Mo-DCs treated with Ad-GFP, Ad-WT, Ad-DN-WT, Ad-P19, Ad-P23, or LPS (2 µg/mL) for 24 h for changes in CD40, CD80, CD86, CD83, HLA-DR and CCR7. Each experimental construct was compared to the empty-vector control, filled histograms, and infected cells were gated on GFP prior to analysis.

Supplemental Table S2. ANOVA test values

	Ad-GFP vs				
	WT	DN-WT	Ad-P19	Ad-P20	Ad-P23
**TNFα**	0.9998	>0.9999	**0.0002**	0.9987	0.1033
**IL-12p70**	0.9985	>0.9999	**<0.0001**	>0.9999	**0.0752**
**IL-10**	0.9926	0.9963	0.9176	0.9406	0.4258
**IL-8**	0.5549	0.9999	**0.0005**	0.8661	**0.0048**
**IFNγ**	0.9996	0.9998	**0.0002**	0.9999	**0.0559**

	Ad-GFP vs			
	WT	DN-WT	Ad-P19	Ad-P23
**Proliferation**	0.3939	0.3638	**<0.0001**	**<0.0001**
**Lysis**	0.3906	0.7983	**<0.0001**	**0.0028**
**Migration**	0.6277	**<0.0001**	**<0.0001**	0.117

	Ad-GFP vs		
**ELISPOT**	Ad-WT	Ad-P19	Ad-P23
	**0.9995**	**0.1658**	**0.0026**

ANOVA analysis of figures 2b, 3a, 3b, 3c and 4b. Yellow denotes p value is significant. Note that two bolded p-values in Ad-P23 close to significant.

Supplemental Figure S3

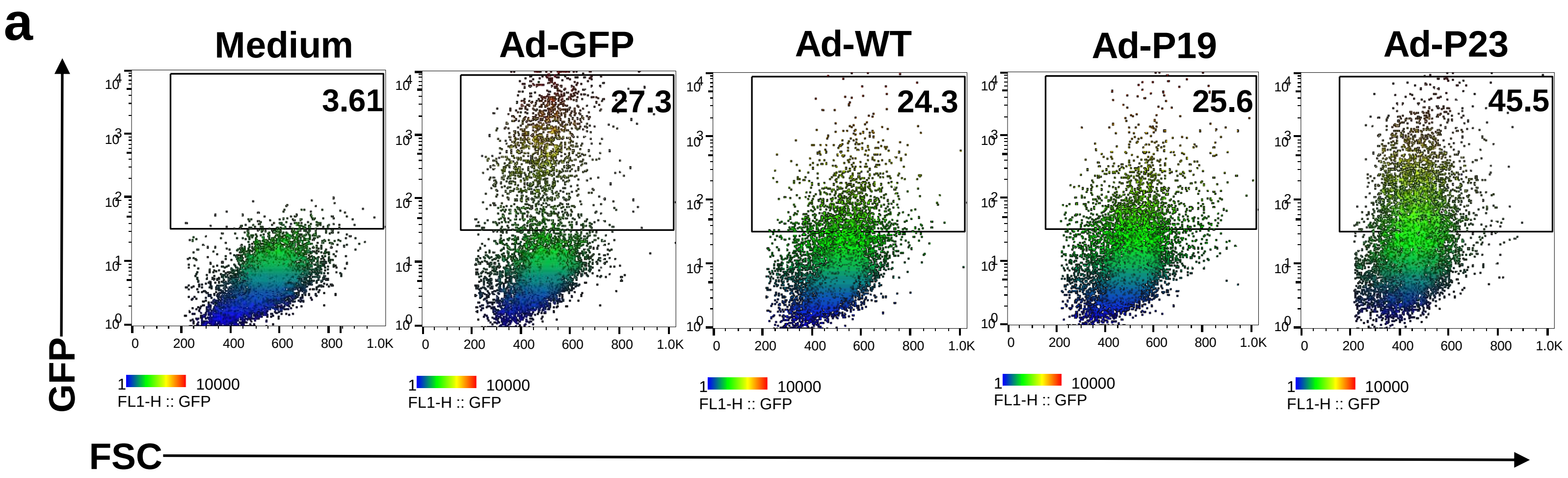

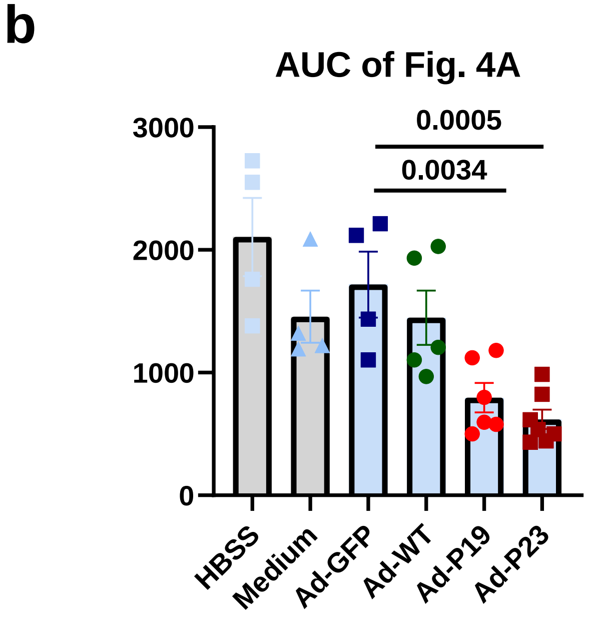

a) GFP expression flow cytometric analysis of bone marrow DCs from animal experiment in Figure 4 transfected with Nothing (medium), Ad-GFP, Ad-WT, Ad-P19, and Ad-P23 (representative figure). b) representation of calculation of area under the curve (AUC) for animals as in “a”. The significance was measured with ANOVA (shown) against Ad-GFP as control). The AUC was performed with animals that survived to the end of the study.

Supplemental Table S3. Paired student t-test values

Day 7	Medium	Ad-GFP	Ad-WT	Ad-p19	Ad-P23
**HBSS vs**	0.227012	0.155804	0.00562	0.000243	0.132482
**Med vs**		0.168829	0.085582	0.126083	0.544817
**AdGFP vs**			0.168767	0.193095	0.720357
Day 10					
**HBSS vs**	0.838925	0.2088	0.191246	0.000179	0.003925
**Med vs**		0.003124	0.111424	0.004858	0.008461
**AdGFP vs**			0.7023	0.021071	0.02903
Day 14					
**HBSS vs**	0.087598	0.120166	0.100367	0.000196	0.000718
**Med vs**		0.450491	0.163527	0.008035	0.002613
**AdGFP vs**			0.245815	0.011623	0.004682
Day 18					
**HBSS vs**	0.064465	0.202463	0.016984	0.000607	0.003114
**Med vs**		0.088198	0.955855	0.009669	0.009832
**AdGFP vs**			0.1784	0.001072	0.001837
Day 21					
**HBSS vs**	0.069485	0.260954	0.061666	0.003163	0.007203
**Med vs**		0.104447	0.671304	0.001028	0.002993
**AdGFP vs**			0.549291	0.004537	0.001536
Day 25					
**HBSS vs**	0.13873	0.055627	0.09018	0.010939	0.00984
**Med vs**		0.259094	0.111343	0.003299	0.001504
**AdGFP vs**			0.22362	0.008242	0.002021
Day 28					
**HBSS vs**	0.225245	0.053565	0.042643	0.01083	0.014383
**Med vs**		0.943765	0.008639	0.022113	0.012809
**AdGFP vs**			0.082507	0.063996	0.017233

Paired student t-test analysis of tumor volume comparisons in Figure 4a. Yellow denotes p value is significant.

Supplemental Table S4. Mutagenesis Primers

Primer name	Sequence
PCDNA3	5’-AATACGACTCACTATAGGGA-3’
	5’-GGACAGTGGGAGTGGCACCTTCCA-3’
DAP12 P7	5’-GTAGACAACCGACCTCTGACC -3
	5’-AGGTCGGTTGTCTACAGCGAC -3’
DAP12 P8	5’-CGCTCATTTGTATTCCGGCCTCTGTGC -3’
	5’- CAGAGGCCGGAATACAAATGAGCGGCC -3’
DAP12 P9	5’-ACCGAGTCGCCTGATC AGGAGCTCCA -3’
	5’-GAGCTCCTGATCAGGCGACTCGGT -3’
DAP12 P10	5’-CGCTCATTTCTCTTCCGGCCTCTGTGT -3’
	5’-CAGAGGCCGGAAGAGAAATGAGCGGCC -3’
DAP12 P11	5’-TCGGATGTCGACAGCGACCTCAAC -3’
	5’-GTGTTGAGGTCGCTGTCGACATCC -3’
DAP12 P17	5’-CAGAGGTCGGTTCGCTACAGCGACCTC-3’
	5’-GTCGCTGTAGCGAACCGACCTCTGACC-3’
DAP12 P18	5’-CAGAGGTCGCATGTCTACAGCCACCTCAA CAC-3’
	5’-TG TTGAGGTGGCTGTAGACATGCGAC CTCTGAC-3’
DAP12 P19	5’-GATGTCTACAGCAA CCTCAACACA CAGAG-3’
	5’-CTGTGTGTTGAGGTTGCTGTAG ACATCCG-3’
DAP12 P20	5’-ACCGAGTCGCCTTGTCAGGAGCTCCAGG-3’
	5’-TGGAGCTCCTGACAAGGCGACTCGGTCTC-3’
DAP12 P21	5’-GTCGGATGTCTGCAGCGACCTCAACACAC-3’
	5’-GTTGAGGTCGCTGCAGACATCCGACCTC-3’
DAP12 P22	5’-GATGTCTACAGCGACTACAACAC ACAGAG-3’
	5’-GCCTCTGTGTGTTGTAGTCGCTGTAGAC-3’
DAP12 P23	5’-GTCTACAGCGACCTCAAAACAC AGAGGC-3’
	5’-CGGCCTCTGTGTTTTGAGGTCGCTG TAG-3’
